# Successful treatment of bilateral empyema with bilateral fistulae using free intercostal muscle flap

**DOI:** 10.1186/s40792-021-01140-8

**Published:** 2021-02-23

**Authors:** Junko Okamura, Naohiro Kobayashi, Takahiro Yanagihara, Shinji Kikuchi, Yukinobu Goto, Yukio Sato

**Affiliations:** Department of General Thoracic Surgery, University of Tsukuba Hospital, 2-1-1 Amakubo, Tsukuba, Ibaraki 305-8576 Japan

**Keywords:** Bilateral empyema with fistula, Empyema, Endobronchial watanabe spigot, Free intercostal muscle flap

## Abstract

**Background:**

Bilateral empyema is rare and can be life-threatening. Few cases have ever been reported about bilateral empyema with fistula on both sides. We herein report a case of bilateral empyema with bilateral fistulae that was treated with a 2-stage operation.

**Case presentation:**

The patient was a 40 year-old man with uncontrolled diabetes mellitus, severe emaciation and remarkably decayed teeth. On his admission, computed tomography showed bilateral pneumothorax and pleural effusion. Thoracentesis revealed a cream-colored purulent pleural effusion from both sides of the pleural cavity. Bilateral empyema with fistulae on both sides due to a ruptured lung abscess was diagnosed. 7 days after his administration, we performed the first surgery. There were 3 fistulae in the right lateral basal segment (S^9^), right posterior basal segment (S^10^), and left posterior basal segment (S^10^). At the first operation, the S^9^ fistula was directly sutured; however, the right S^10^ fistula could not be closed because the surrounding tissue was fragile. The left lung fistula was deep and crater-shaped; it was closed with the suturing of a plugged free muscle flap. At the second operation, the right S^10^ fistula was closed with the superimposition of a pedicled intercostal muscle flap.

**Conclusion:**

Patients with bilateral empyema tend to be with poor general condition and, therefore, less invasive treatments are required initially. Closure of fistulae is an essential process for the treatment of empyema with fistulae. We could manage the fistulae using several techniques with 2-stage operation. Although the efficacy of using a free intercostal muscle flap to close the fistula has not been adequately verified, it is simple and less invasive and, thus, might be a useful option in cases where the patient is too ill to undergo a more invasive operation or when the surgical approach should be done in a short time

## Background

Empyema is a critical infectious disease that has a reported mortality of 6 to 24% [1, 2]. Bilateral empyema is uncommon and is severely life-threatening [2–4]. We herein describe a case of bilateral empyema with bilateral fistulae treated successfully with a 2-stage operation.

## Case presentation

**Fig. 1:**
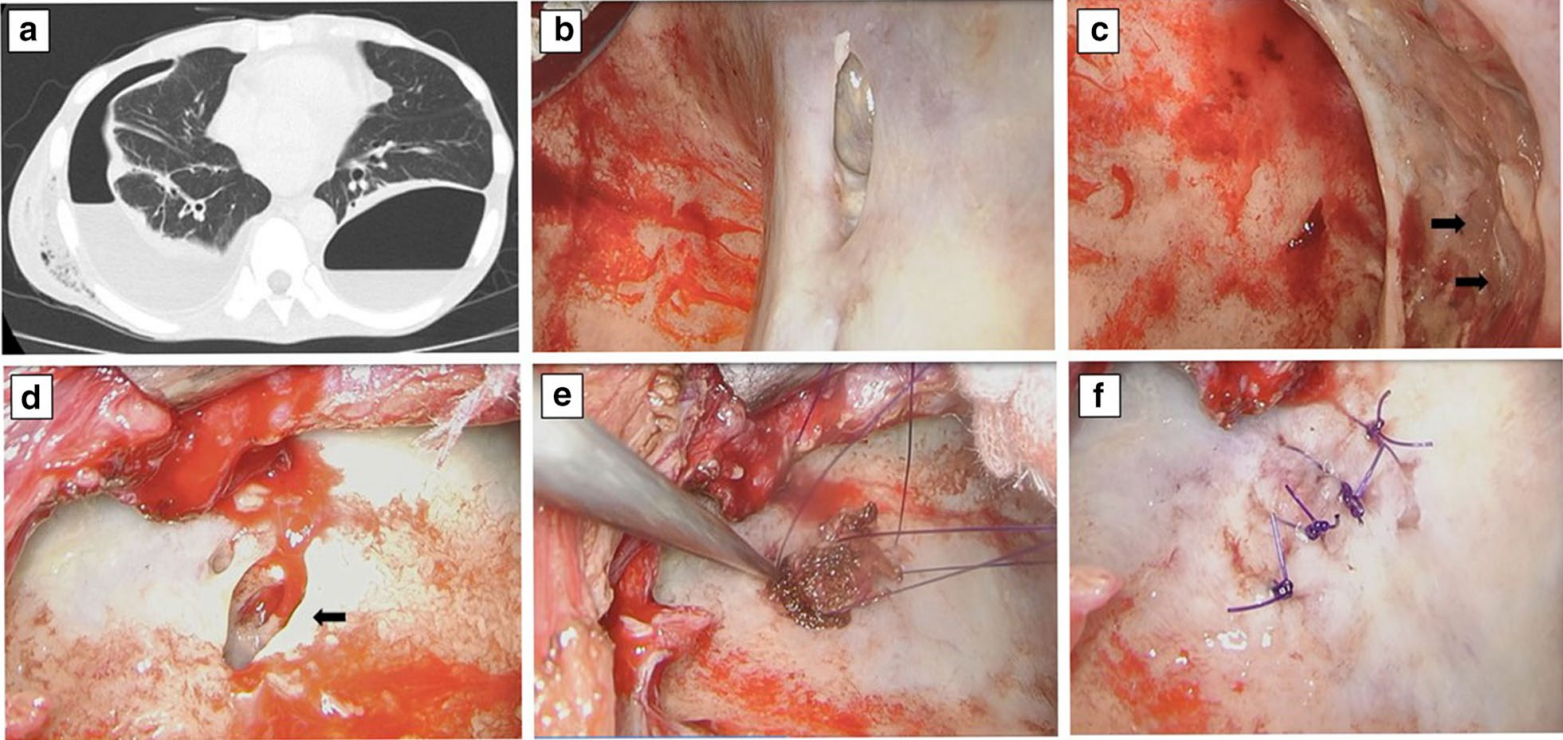
**a** CT on admission showed bilateral pneumothorax and pleural effusion. The intraoperative findings at the first surgery are shown: **b** fistula in the right lateral segment (S^9^), **c** fistula in the right posterior segment (S^10^) (black arrow), **d** fistula in the left posterior basal segment (S^10^) (black arrow), **e** free intercostal muscle flap plugged into the left fistula, **f** left S^10^ fistula sutured after plugging with the free intercostal muscle flap

A 40-year-old man was admitted to hospital exhibiting fatigue, weight loss of 10 kg, and dyspnea on effort. Physical examination showed severe emaciation (body mass index 15.1) and remarkably decayed teeth. He could not walk due to malnutrition. Laboratory data showed glucose tolerance failure (HbA1c 14.3%, blood glucose level 527 mg/dL) and hypoalbuminemia (serum albumin 2.4 g/dL). C-reactive protein level was 0.25 mg/dL, and white blood cell count was 5000 mm^3^. Computed tomography showed bilateral pneumothorax and pleural effusion (Fig. 1a). Thoracentesis revealed a cream-colored purulent pleural effusion from both sides of the pleural cavity. A diagnosis was made of bilateral empyema with bilateral fistulae due to a ruptured lung abscess and antibiotics (sulbactam sodium/ampicillin sodium) was introduced. He was transferred to our hospital for further treatment. Chest drainage tubes were inserted into both sides of the pleural cavity. Bacterial culture of the right-side pleural effusion showed streptococcal species but no pathogenic species were found in the left pleural effusion. Antibiotics was stopped because laboratory data showed probable antibiotics-induced liver damage. Intensive insulin therapy and dietary therapy (energy control meal, 1600 kcal/day) were introduced for diabetes mellitus treatment.

Seven days after the patient was admitted to our hospital, we performed the first operation under thoracoscopy. The operation was started on the right side because the air leakage was predominant on that side. In the right pleural cavity, the infected pleura was white and thick, and visceral pleural crater-shaped fistulae were observed. The infected fluid and material were removed, and the pleural space was cleansed with saline. On the right side, two fistulae were observed, one in the right lateral basal segment (S^9^) (Fig. 1b) and the other in right posterior basal segment (S^10^) (Fig. 1c). The sizes of the fistulae were approximately 2 cm and 3 cm, respectively. During a water-sealing test, the water reversed though the double-lumen endobronchial tube, so water-sealing test was not repeated in order to prevent lung damage induced by washing the pleural cavity. The S^9^ fistula was directly sutured using 3–0 absorbable, monofilament sutures; however, the S^10^ fistula was a relatively large fistula and difficult to close under thoracoscopy being located near the costophrenic angles. In addition, the surrounding tissue was fragile because of inflammation, and there was a possibility that suturing this tissue would cause further air leakage. A chest drainage tube was placed in the right pleural cavity locating the tip of catheter near the S^10^ fistula. Washing of the pleural cavity, removal of the infected pleural tissue, and drainage of the left side were sequentially performed. On the left side, a crater-like fistula, approximately 1.5 cm in size, was present in the left posterior basal segment (S^10^) (Fig. 1d) and given its deep cavitied shape, the fistula was deemed to be difficult to close with direct suturing. Therefore, free intercostal muscle tissue, located around the thoracoscopic port and measuring about 2 cm, was harvested. We filled and plugged the fistula using the flap (Fig. 1e). Then, the free intercostal muscle flap and lung tissue around the fistula were sutured together using 3–0 absorbable, monofilament thread (Fig. 1f). By the end of the operation, the chest drainage unit showed air leakage only within the right pleural cavity.

**Fig. 2:**
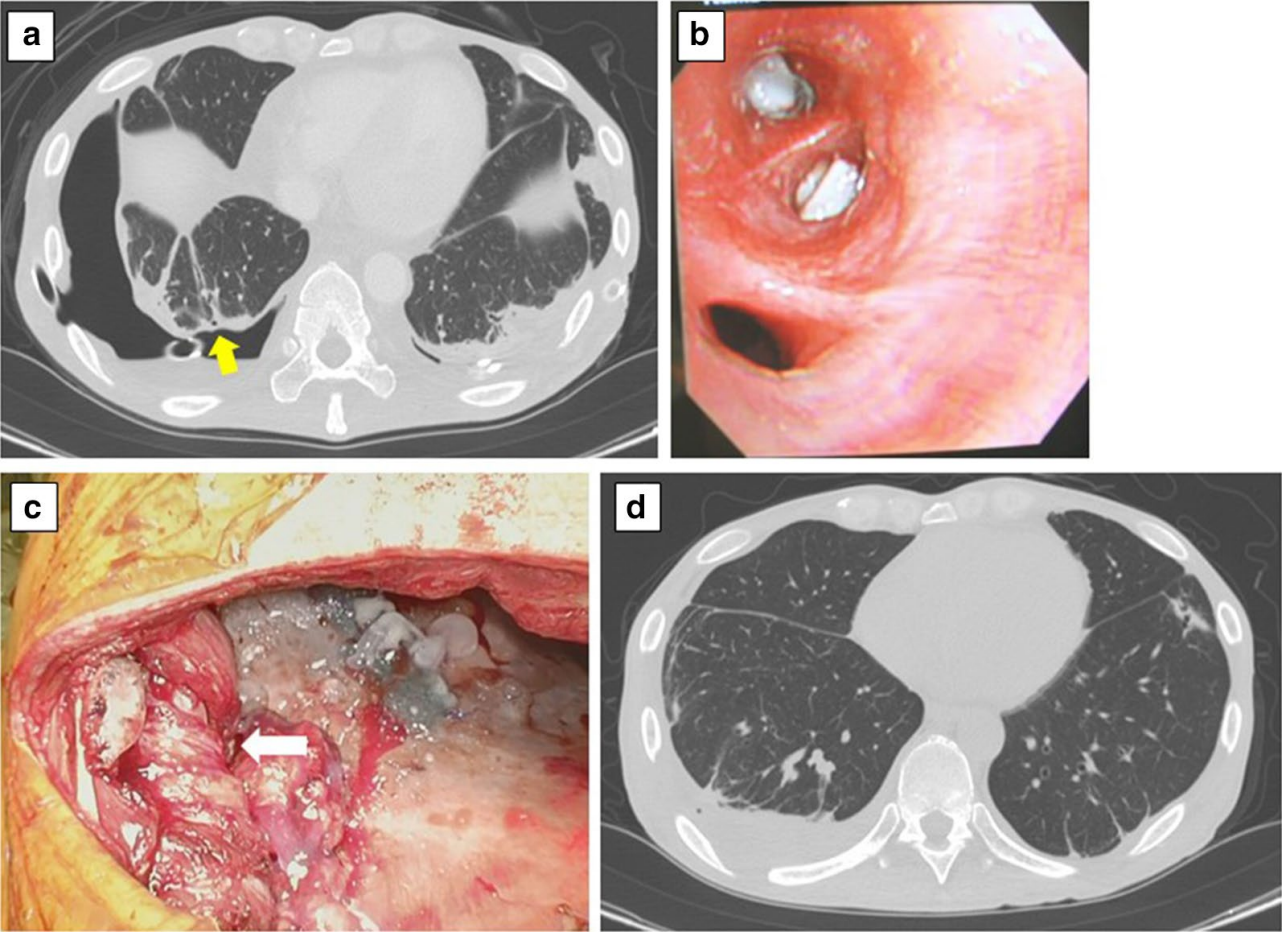
**a** CT after the first operation revealed a bronchopleural fistula in the right pleural cavity. The right B^10^c was thought to be the responsible bronchus (yellow arrow). **b** Endobronchial Watanabe spigots were inserted into the right B^10^b and B^10^c bronchi. **c** The right S^10^ fistula was superimposed and closed using a pedicled intercostal muscle flap (white arrow). **d** CT after the second operation showed that both sides of the lung expanded without residual pleural space

Postoperative CT showed almost full expansion of the left lung and a bronchopleural fistula of the right B^10^ bronchus (Fig. 2a). With the patient under conscious sedation (midazolam), forceps were used to insert endobronchial Watanabe spigots into the right B^10^b and B^10^c bronchi (Fig. 2b), following which, the air leakage of the right pleural cavity tended to decease but was refractory. Therefore, a second operation for closure of the right S^10^ bronchopleural fistula was carried out 14 days after the first operation. At that time, the patient’s general condition had improved due to insulin therapy, dietary therapy and rehabilitation and he was able to walk with walking frame. The right ninth rib was removed, and a pedicled ninth and tenth intercostal muscle flap was created. The pedicled intercostal muscle flap was superimposed over the S^10^ fistula with anchor sutures (Fig. 2c). Antibiotics (meropenem) was administered for seven days following the second operation. After the second operation, the air leakage stopped completely, and the right lung expanded fully (Fig. 2d). Right pleural effusion was culture negative, so the chest drainage tube was removed seven days after the second operation. Further rehabilitation, dietary therapy and insulin therapy were continued after the second operation. His body weight increased from 40.7 kg at the time of admission to 47.1 kg. The patient was transferred to another hospital for continuation of rehabilitation.

## Discussion

Empyema is typically unilateral, while bilateral empyema is an uncommon, life-threatening condition that can result from pneumonia, septic emboli, or mediastinitis [5]. According to previous reports [2–4, 6], the characteristics of adult patients with bilateral empyema have much in common with those who have alcohol addiction, diabetes mellitus, a history of heavy smoking, poor nutritional status, and significant tooth decay. The conventional procedures for treatment of empyema with fistulae are direct suturing, superimposition of the pedicled muscle flap on the fistula, and plugging of the responsible bronchi with materials such as an endbronchial Watanabe spigot.

In the current case, consistent with previous reports, the patient had many complications and health problems such as uncontrolled diabetes mellitus, decayed teeth, poor nutritional status and could not walk at the time of admission. His poor general physical condition necessitated a less invasive initial surgery. At the first operation, the right S^10^ fistula was in a difficult location for suturing under thoracoscopy and relatively large for directly suturing. In addition, direct suturing would possibly have caused further air leakage because the lung tissue around the fistula appeared to be fragile. We considered using pedicled intercostal muscle flaps to close the right S^10^ fistula, however, the patient’s poor general condition precluded the use of excessive surgical interventions. Thus, we planned a 2-stage surgery in which the fistula would be addressed after his general condition had improved. By the time of the second operation, the right S^10^ fistula had actually decreased in size since the time of the first operation and lung tissue around the fistula showed some reduction of fragility, presumably owing to control of the infectious source and the patient’s improved general condition. This improvement in condition allowed for the successful closure of the S^10^ fistula using pedicled intercostal muscle flaps. The left S^10^ fistula was crater-shaped and deeply cavitied. Direct suturing would have been a less invasive approach but it is not suitable for closure of deep fistula while remaining airtight. Harvesting the free intercostal muscle flap did not require additional skin incision and the flap was quickly created with minimal invasiveness. In addition, it is easily deformable material in response to a fistula shape. To strengthen lung tissue around the fistula and to control the infectious source, we thought plugging the fistula with the free intercostal muscle flap may be effective. We had also considered the use of a pedicled intercostal muscle flap at a later stage in case the free intercostal muscle flap failed to close the left S^10^ fistula. However, we were able to successfully close the fistula using the free intercostal muscle flap, which plugged the fistula in an airtight manner and resulted in complete stoppage of the air leakage. For unwell and undernourished patients, this method has advantages in terms of its simplicity and being less invasive than open-window thoracostomy or pedicled omentum flap interposition. Furthermore, our 2-stage operation strategy, in which more invasive procedures were planed after the patient’s general condition had improved, succeeded in complete control of the air leakage. In our case, open-window thoracostomy would have been one of the potentially effective surgical strategies. Had open-window thoracostomy been selected in the right pleural cavity at the time of first operation, the time taken to control the infectious source in the right side would likely have been reduced. However, at the time of the first operation on the right side, the condition of the left pleural cavity was uncertain, as such there was a possibility that the left pleural cavity may also have required open-window thoracostomy and bilateral open-window thoracostomy carries an increased risk of respiratory failure. In our case of bilateral fistulae, successful closure of the right fistula was achieved in the second operation 14 days after the first operation without open-window thoracostomy. Therefore, free intercostal muscle flap can be a useful approach for initial surgery for bilateral empyema with fistula.

Although free muscle flap does not have a blood supply, several reports have suggested that the use of a free graft patch, such as pericardial fat, periosteum, or costal cartilage, could be effective for closing bronchopleural fistulae and stopping alveolar air leakage [7–10]. This is the first report of empyema-associated fistula resulting from ruptured lung abscess to be successfully treated with free intercostal muscle flap. Although the long-term viability of free intercostal muscle flaps has not yet been verified and the extrapolation of this strategy using free intercostal muscle flap needs more accumulation of cases, we found that covering the fistula with a free intercostal muscle flap in an airtight manner was effective.

## Conclusion

We successfully treated bilateral empyema with bilateral fistulae using several techniques. Because the general physical condition of a patient with bilateral empyema tends to be poor, less invasive therapeutic and surgical options are preferable at first. Using a free intercostal muscle flap to aid the closure of a deep fistula might be an effective treatment option in cases where the patient is too ill to withstand a more invasive operation.

## Data Availability

Not applicable.

## Abbreviations

Right S^9^: Right lateral basal segment
Right S^10^: Right posterior basal segment
Left S^10^: Left posterior basal segmen
HbA1c: Hemoglobin A1c
CT: Computed tomography
